# Genome-wide analysis of circular RNAs in bovine cumulus cells treated with BMP15 and GDF9

**DOI:** 10.1038/s41598-018-26157-2

**Published:** 2018-05-21

**Authors:** Yao Fu, Hao Jiang, Jian-Bo Liu, Xu-Lei Sun, Zhe Zhang, Sheng Li, Yan Gao, Bao Yuan, Jia-Bao Zhang

**Affiliations:** Department of Laboratory Animal Science, College of Animal Sciences, Jilin University, Changchun, 130062 Jilin, P.R. China

## Abstract

Circular RNAs (circRNAs) are important members of the non-coding RNA family, and those relating to animal physiologies have been widely studied in recent years. This study aimed to explore the roles of circRNAs in the regulation of follicular development. We constructed four bovine cumulus cell cDNA libraries, including a negative control group (NC) and groups treated with BMP15, GDF9 and BMP15 + GDF9, and we sequenced the libraries on the Illumina HiSeq Xten platform. We identified 1706 circRNAs and screened for differential circRNA expression. We conducted a bioinformatics analysis of these circRNAs and screened for differential circRNAs. Functional annotation and enrichment analysis of the host genes showed that the differential circRNAs were related to locomotion, reproduction, biological adhesion, growth, rhythmic processes, biological phases and hormone secretion. According to the differential expression of circRNA between groups, there were 3 up-regulated and 6 down-regulated circRNAs in the BMP15 group as well as 12 up-regulated and 24 down-regulated circRNAs in the GDF9 group. Co-addition of both BMP15 and GDF9 resulted in 15 up-regulated and 13 down-regulated circRNAs. circ_n/a_75,circ_12691_1 and circ_n/a_303 were altered in both the BMP15 and GDF9 groups as well as in the BMP15 + GDF9 combination group. We focused on these three circRNAs because they were potentially associated with the additive effect of BMP15 and GDF9. Quantitative PCR analysis showed that the expression levels of these three circRNAs were consistent with the sequencing results. In addition, the target miRNAs of circ_n/a_75 and circ_n/a_303, miR-339a, miR-2400 and miR-30c, were down-regulated in the experimental group, which was in contrast to the circRNAs trend. These findings demonstrated that BMP15 and GDF9 may regulate the target gene through circRNA, as a miRNA sponge, in order to regulate the status of bovine cumulus cells and affect follicular development.

## Introduction

As their name implies, circular RNAs (circRNAs) are a class of closed circular RNA molecules predominantly produced by the alternative shearing of pre-mRNA. These molecules are abundant in the eukaryotic transcriptome and serve as important members of the non-coding RNA family^1,2^. CircRNAs were found in viroids, a RNA virus^3^, as early as 1976, and they have been long thought to be a by-product of misincorporation. CircRNAs are generated by back-splicing from directly linked 5′ and 3′ termini of linear RNA^4^, and they are conserved in different species and exhibit differential tissue expression. CircRNAs are not sensitive to nucleases because of their circular structure, making them more stable than linear RNAs. These properties make circRNAs promising tools for the development of novel diagnostics, and their tremendous potential for treatment is of paramount importance^5,6^.

As a new class of RNA molecules, circRNAs are derived from a variety of sources and are classified the following five ways depending on how they are synthesized: lariat-driven circularization, intron-pairing-driven circularization, circular intronic RNAs, RNA-binding proteins and trans-factor driven circularization. Notably, other possible models of alternative circularization also exist^6,7^. According to the position of the gene in the genome, a gene can be divided into (1) sense, (2) antisense, (3) exonic, (4) intronic, and (5) intergenic regions.

Some studies have shown that circRNAs exert important regulatory functions at both the transcriptional and post-transcriptional level. For example, circRNAs can act as competing endogenous RNAs (ceRNAs), bind intracellular miRNAs, and block the suppression of miRNA target genes^8^. The first circRNA that was found to act as an miRNA sponge was CDR1as, which has more than 70 conserved miRNA-7-binding sites^9^. In addition, circ-SRY, which is specifically expressed in mouse testis, is a circRNA with 16 miRNA-138-binding sites^10^. CircRNAs have a variety of biological functions, playing roles in alternative splicing and translation as well as in parental gene expression regulation, and a few other circRNAs are directly translatable^7,11–13^. Although numerous studies have found that circRNAs are widely expressed in humans and mice, only a few studies have been conducted in other mammals, especially in cattle.

Mammalian follicles are composed of oocytes and granulocytes, and follicle development utilizes a delicate communication process between the oocyte and its surrounding somatic cells. Granulosa cells are the most important cell type in the physiological processes of ovarian follicle development, differentiation, ovulation, luteinization and atresia. Oocyte can promote the proliferation and differentiation of granulosa cells, and the granulosa cells quality can also affect oocyte maturation. In mice lacking the granulocyte-oocyte connexin, GK-37, oocyte meiosis maturation is blocked^14^. Additional studies have shown that granulosa cells in the ovary are involved in the regulation of follicular growth and development^15^, meiosis^16^ and overall transcriptional activity^17,18^.

Follicular development, oocyte maturation, cumulus expansion, and ovulation all rely on crosstalk between the oocyte and granulosa cells. Granulosa cells are structurally and functionally divided into two types as follows: those distributed in the follicular wall (mural granulosa cells, MGCs) and those distributed around the oocyte (cumulus cells, CCs). Cross-talk between CCs and oocytes has a direct impact on follicular development and oocyte maturation.

Oocytes are potent stimulators of granulosa and cumulus cell proliferation in parietal layers while simultaneously inhibiting their apoptosis and death. Bone morphogenetic protein 15 (BMP15) and growth differentiation factor 9 (GDF9) are two growth factors secreted by oocytes. GDF9 and BMP15 bind to receptors in CCs, inducing a cascade reaction of downstream genes that affects their proliferation and apoptosis, thus regulating follicle development and oocyte maturation^19^.

BMP15 and GDF9, TGF-β superfamily members mainly derived from follicle oocytes, are highly homologous in structure and are expressed in the ovary, and increasing evidence has shown synergy between these two proteins^20–24^. GDF9 and BMP15 are important regulators of follicular growth and ovarian function. GDF9 and BMP15 regulate the differentiation, proliferation and function of local ovarian cells via autocrine and paracrine mechanisms, and they play important roles in follicular growth, atresia, ovulation, fertilization and reproductive maintenance. Studies have shown that the addition of GDF9 or BMP15 to cultured oocytes *in vitro* can improve blastocyst and embryo transfer rates^25–27^.

To explore the mechanisms underlying the effects of BMP15 and GDF9 on bovine CC proliferation and apoptosis, we investigated the roles of circRNAs in this process and the physiological mechanism underlying follicles from the non-coding RNA perspective. Herein, BMP15 and GDF9 were added to CCs cultured *in vitro*. We used 4 nM HCL as a negative control group (NC) (4 nM HCL as BMP15 solvent). The full circRNA expression profile was obtained by deep RNA sequencing (RNA-seq). Differential circRNAs related to the additive effect of BMP15 and GDF9 were screened and validated by qRT-PCR and functional analysis, revealing a new approach to further study the physiological mechanisms underlying follicular development.

## Results

### Identification and characteristics of circular RNA expression profiles in bovine cumulus cells treated with BMP15 and GDF9

In this study, BMP15 and GDF9 were added to bovine CCs cultured *in vitro* to identify the expression of circRNAs under various conditions. The concentrations of BMP15 and GDF9 added alone were 100 ng/μl and 200 ng/μl, respectively, and their co-addition concentrations were 50 ng/μl and 100 ng/μl, respectively. Different libraries were pooled according to the target machine data volume and sequenced on the Illumina HiSeq platform. Raw data were filtered, and linker sequences and low-quality reads were removed to obtain high-quality clean data. After sequencing the quality control, a total of 76.92 Gb of clean data were obtained. The numbers of reads in each group were 54,893,253 (NC), 65,396,545 (100 ng/μl BMP15), 73,994,644 (200 ng/μl GDF9), and 62,454,119 (50 ng/μl BMP15 + 100 ng/μl GDF9). The clean data were aligned to the specified reference genome to obtain the mapped data. Statistical analysis showed that the efficiencies of the sample and reference genomes ranged from 99.97% to 99.98%, which indicated that data utilization was normal and that the selected reference genome met the needs of the subsequent analysis (Table 1).

**Table 1:** Statistics of sample sequencing data.

Samples	Read Number	Base Number	GC Content	Total Reads	Mapped Reads
NC	54,893,253	16,445,580,268	49.60%	109,786,506	109,757,412 (99.97%)
BMP15	65,396,545	19,580,030,880	49.21%	130,793,090	130,762,146 (99.98%)
GDF9	73,994,644	22,176,945,244	49.20%	147,989,288	147,951,550 (99.97%)
BMP15 + GDF9	62,454,119	18,717,550,392	49.79%	124,908,238	124,882,940 (99.98%)

Read number: total number of pair-end reads in clean data; base number: total base number in clean data; GC content: percentage of total G and C bases in the clean data; total reads: number of clean reads based on a single end; mapped reads: number of reads aligned to the reference genome and percentage in clean reads.

**Figure 1:**
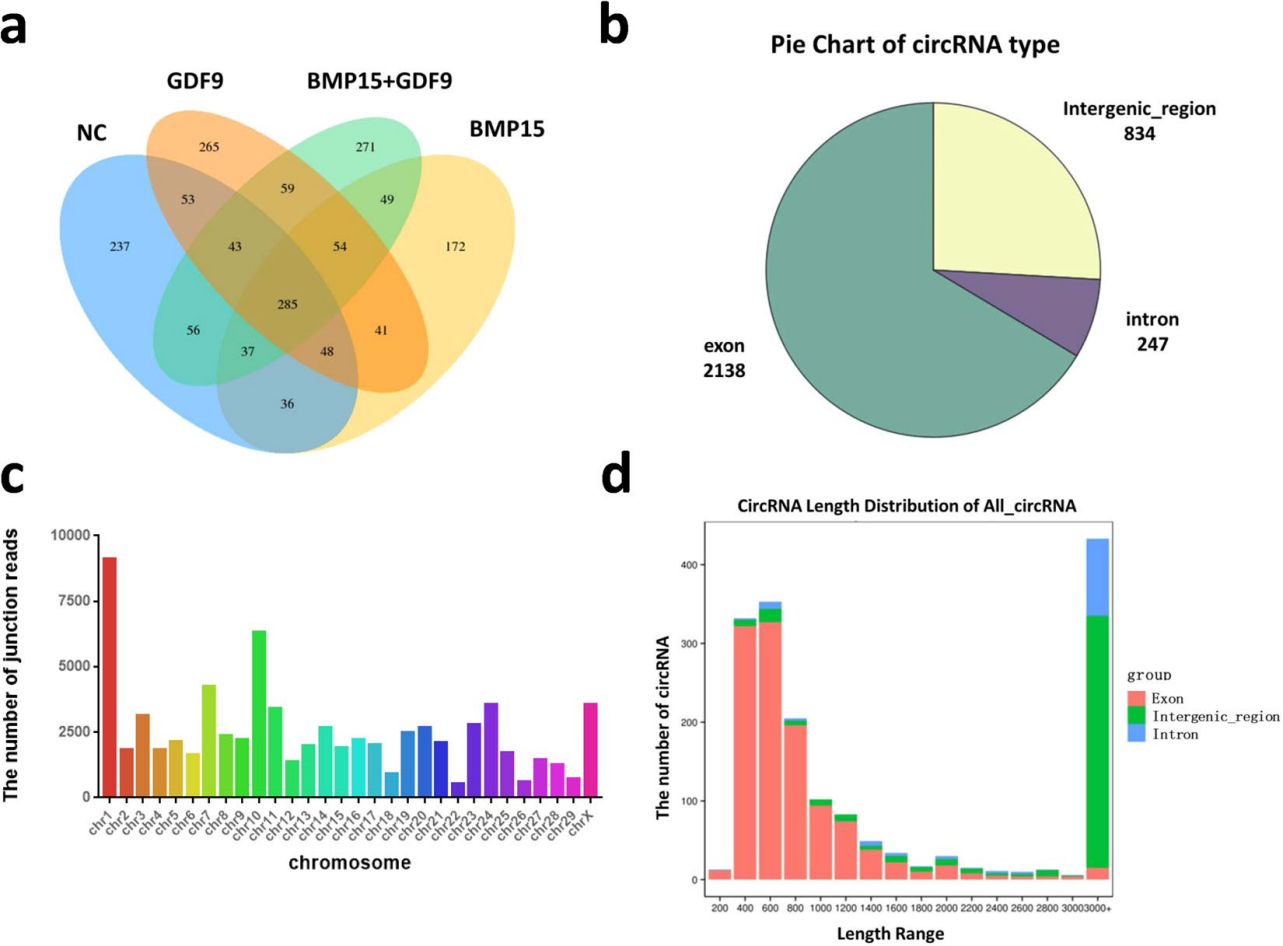
Identification and characteristics of circRNAs. (**a**) The number of circRNAs expressed in each sample. (**b**) CircRNA source profiles for circRNAs. (**c**) CircRNA chromosomal profiles for all circRNAs. (**d**) Sequence length distributions.

We used CIRI and find-circ software to predict the circRNAs, and a total of 1706 circRNAs were detected. The numbers of circRNAs expressed in each sample were displayed by a Venn diagram (Fig. 1a).

CircRNAs have different genomic distributions, as they can originate from exons, introns, or intergenic regions. Therefore, the distributions of the detected circRNAs in the genome are shown in the pie chart. According to the database, most of the circRNAs originated from exonic regions followed by intergenic regions and intron regions (Fig. 1b). Next, we counted the distributions of the circRNAs on different chromosomes, revealing that chromosomes 1 and 10 produced the most circRNAs (Fig. 1c). The distributions of the circRNA lengths in each sample showed that those ranging from 200–2000 bp in length mainly originated from exons, while those longer than 3000 bp mainly originated from intergenic regions (Fig. 1d).

### Differentially expressed circRNAs in bovine cumulus cells treated with BMP15 and GDF9

**Figure 2:**
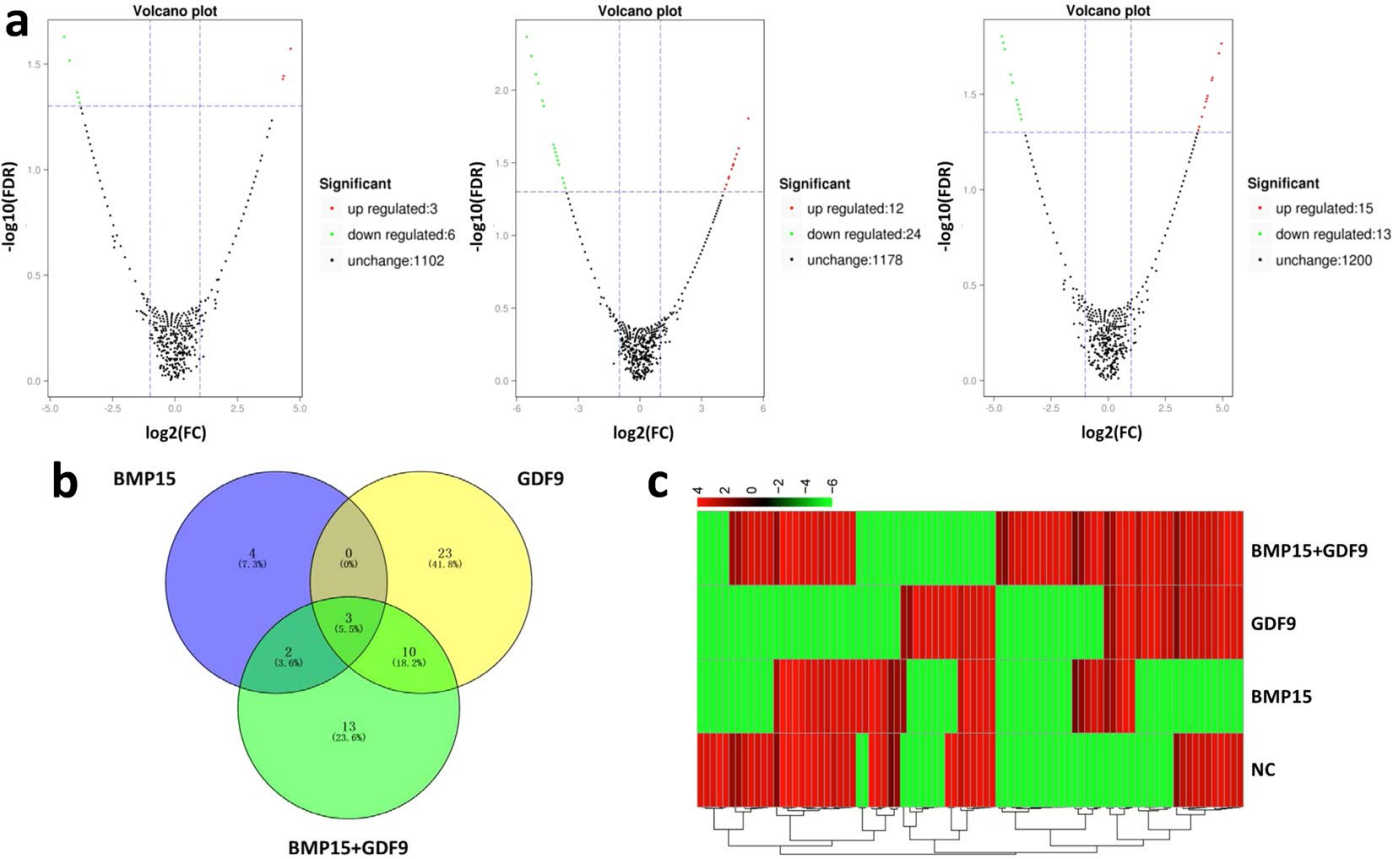
Differential expression of circRNAs in cumulus cells. (**a**) Volcano map of different circRNAs for each group. Each point in the volcano represents a circRNA, and the abscissa represents the logarithm of the fold change (FC) of a circRNA in two samples. Higher absolute values are correlated with larger differences. The ordinate represents the negative logarithm of the false discovery rate (FDR). Down-regulated circRNAs are represented in green, and up-regulated circRNAs are shown in red. Black dots represent circRNAs that are not significantly different. (**b**) Venn diagram of circRNA expression differences among the groups. (**c**) Hierarchical clustering of partially differentially expressed circRNAs. ‘Red’ indicates high relative expression, and ‘green’ indicates low relative expression.

When testing the differential expression of the circRNAs, fold changes (FCs) greater than or equal to 2 and false discovery rates (FDRs) less than 0.05 served as the screening criteria. The FC indicated the ratio of expression levels between two samples (groups), and the FDR was used as the key screening indicator of differentially expressed circRNAs. The volcano plot visually shows the relationship between the FDRs and FCs of all circRNAs, allowing the quick evaluation of circRNA expression variability between two samples and its statistical significance (Fig. 2a, Supplemental Tables S1–S3).

Addition of BMP15 resulted in 3 up-regulated and 6 down-regulated circRNAs, and GDF9 treatment resulted in 12 up-regulated and 24 down-regulated circRNAs. Co-addition of both BMP15 and GDF9 resulted in 15 up-regulated and 13 down-regulated circRNAs. The numbers of circRNAs differentially expressed between the groups are shown by Venn diagram. Among these circRNAs, 3 were altered in all three experimental groups, showing the same trend. BMP15 and GDF9 had an additive effect on these circRNAs (Fig. 2b).

Next, we conducted hierarchical clustering analysis of the differentially expressed circRNAs, and clustering among the four groups of samples is shown in Fig. 2c.

### Functional annotation and enrichment analysis of circRNA host genes

**Figure 3:**
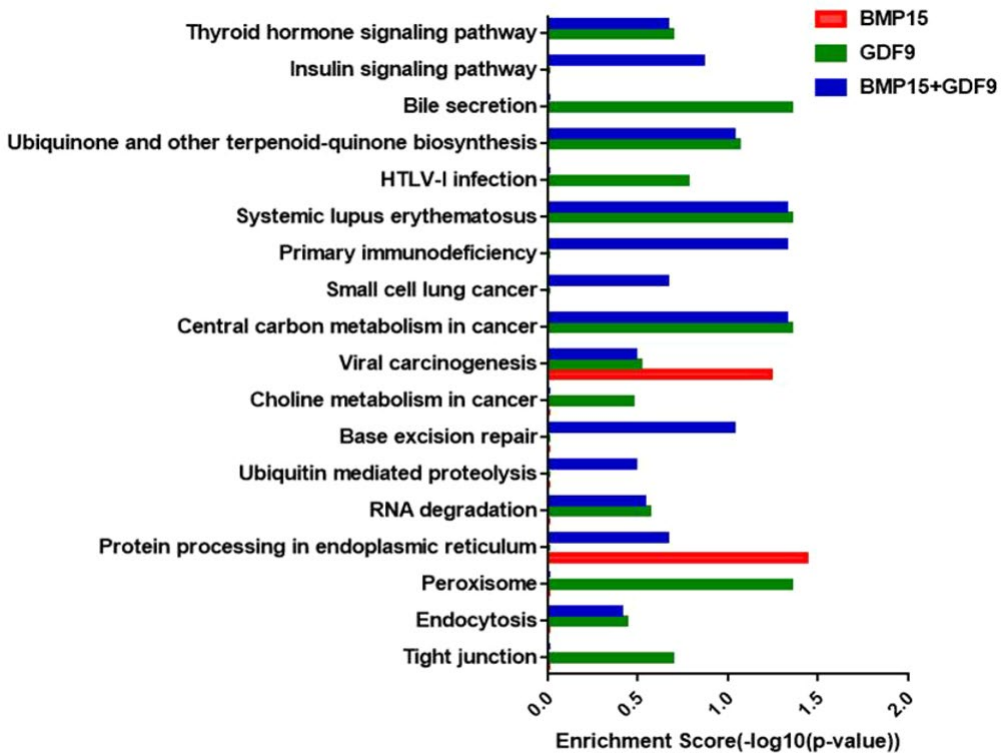
Functional annotation and enrichment analysis of host genes. KEGG pathway analysis of host genes of the differential circRNAs.

To explore the mechanisms underlying the effects of BMP15 and GDF9 on CCs, we performed functional annotation analysis on the genes producing differentially expressed circRNAs. Gene ontology (GO) analysis revealed that numerous host genes showed strong relationships with biological processes, cellular components, and molecular functions. These biological processes included locomotion, reproduction, biological adhesion, growth, rhythmic processes, biological phases and hormone secretion (Supplemental Figures S1–S3). Different gene products coordinate with each other to perform biological functions, and pathway annotations of circRNA host genes provide a better understanding the function of these genes. Kyoto Encyclopedia of Genes and Genomes (KEGG) analysis showed that 18 pathways were related to circRNAs (Fig. 3, Supplemental Tables S4–S6).

### Validation of circRNA expression and target gene prediction

**Figure 4:**
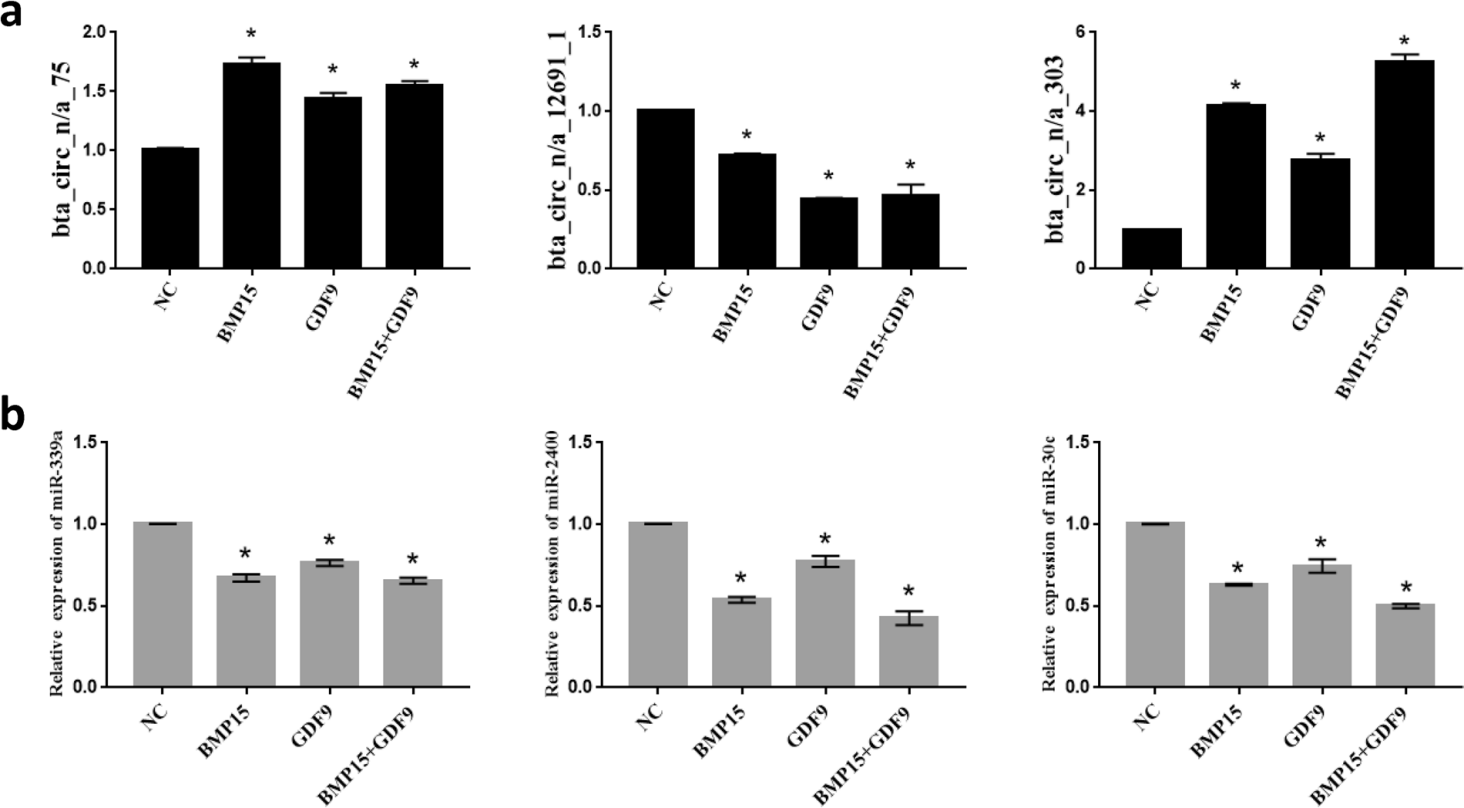
qPCR validation of circRNAs and their target miRNAs. (**a**) Expression levels of circ_n/a_75, circ_12691_1 and circ_n/a_303. (**b**) Expression levels of the miR-339a, miR-2400 and miR-30c. All experiments were repeated at least three times. The data are shown as the means ± SD. Statistical significance was analyzed by one-way ANOVA, and P < 0.05 was considered significant. *Denotes a statistically significant difference (P < 0.05).

By analyzing the sequencing data, we found 3 circRNAs (circ_n/a_75, circ_n/a_303 and circ_12691_1) that were different among the BMP15, GDF9 and co-addition groups. We speculated that these 3 circRNAs may associate with BMP15 and GDF9 to regulate the proliferation and apoptosis of CCs. Therefore, we verified the expression levels of these 3 circRNAs in CCs after BMP15 and GDF9 treatment by quantitative PCR (qPCR), and the results were highly consistent with those of RNA-seq. circ_n/a_75 and circ_n/a_303 were up-regulated in all three treatment groups, while circ_12691_1 expression was down-regulated in all three treatment groups,(Fig. 4a). To explore the ceRNA circRNA molecules, the circRNA-miRNA-mRNA networks were predicted, revealing miR-339a to be a miRNA target of circ_n/a_75 and miR-2400 and miR-30c to be miRNA targets of circ_n/a_303. We used qRT-PCR to verify the expression levels of these three miRNAs (miR-339a miR-2400 and miR-30c) in each treatment group, revealing that the predicted miRNAs of circRNAs in the experimental groups were significantly lower than those in the control group, which was opposite from the corresponding circRNA trend (Fig. 4b).

**Figure 5:**
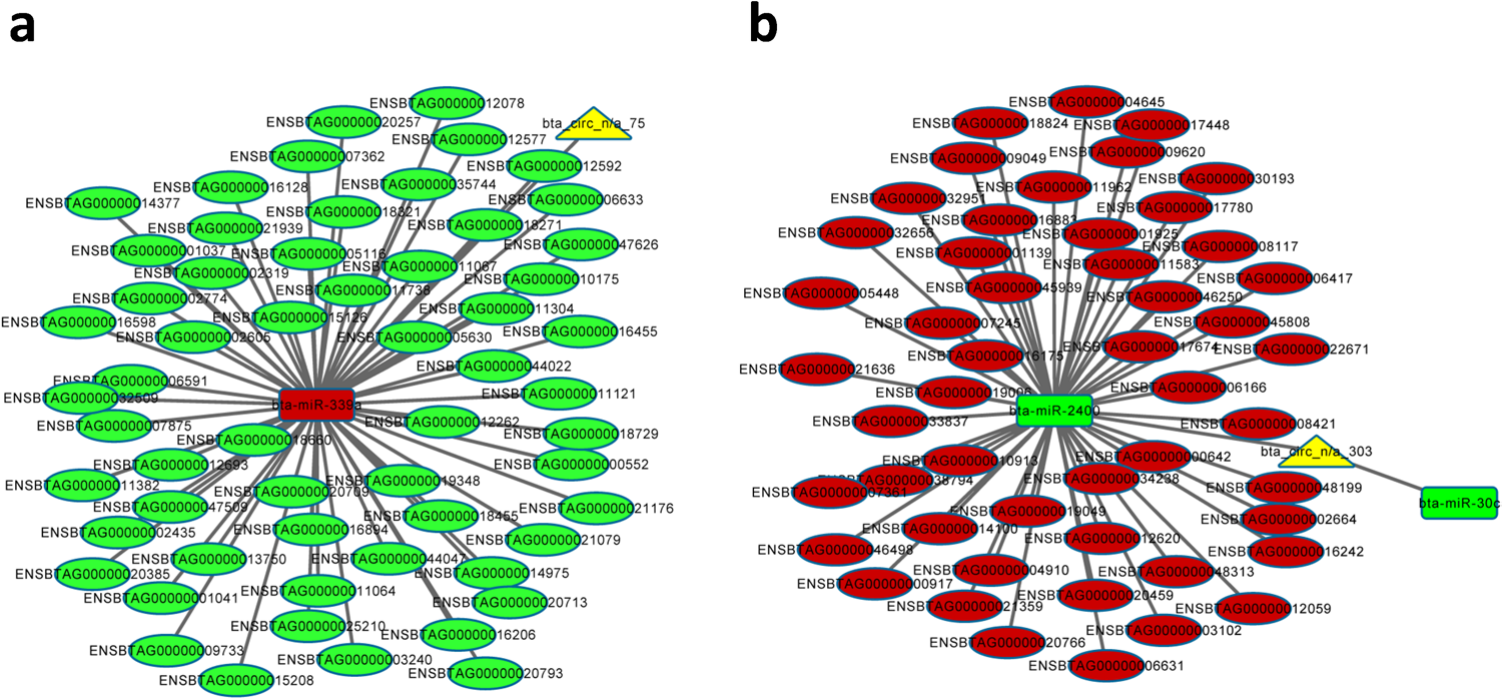
The target miRNA-mRNA network was predicted biomathematically. (**a**) Circ_n/a_75 circRNA-miRNA-mRNA network. (**b**) Circ_n/a_303 circRNA-miRNA-mRNA network.

**Figure 6:**
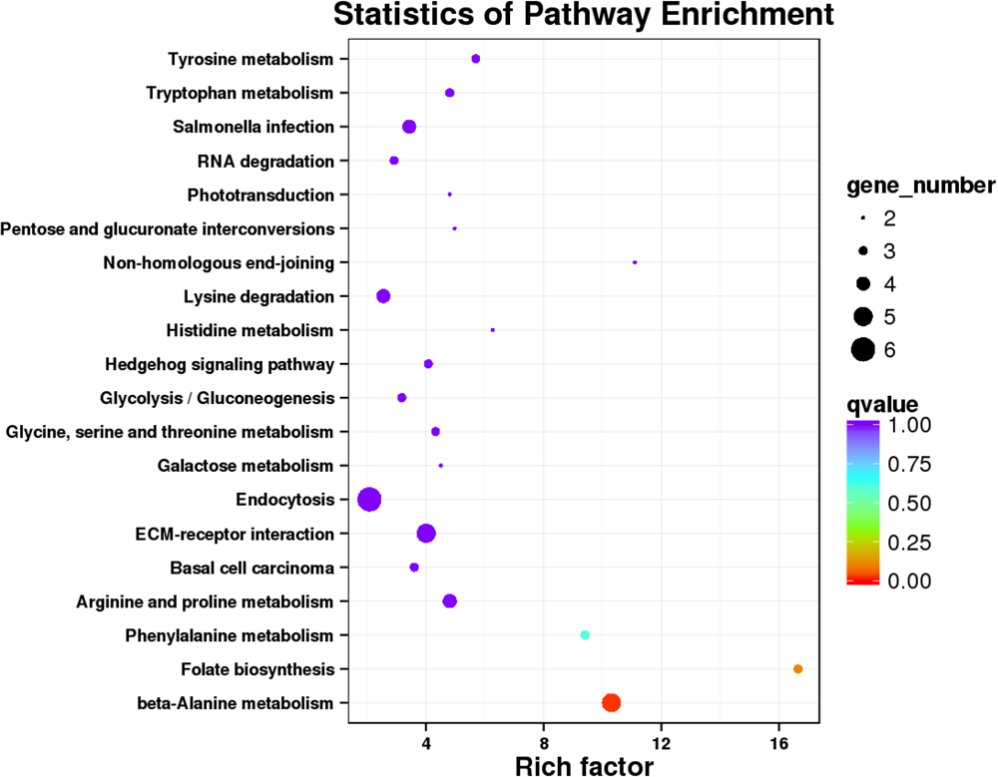
KEGG pathway enrichment analysis of the predicted target genes of miR-339a and miR-2400.

We next elucidated the integrated circRNA-miRNA-mRNA networks (Fig. 5a,b). CircRNA targets are known to regulate miRNAs by acting as sponges, thereby affecting the function of downstream target genes. Therefore, we analyzed the target genes of the abovementioned miRNAs. miR-339a had 58 target genes, and miR-2400 had 50 target genes. Annotation of the target genes showed a strong correlation with tyrosine metabolism, tryptophan metabolism, beta-alanine metabolism, endocytosis and other pathways (Fig. 6).

## Discussion

Throughout the oogenesis process, oocytes are coupled to surrounding granulosa cells via gap junctions, and granulosa cells are also related to each other. This highly specific membrane junction mediates the transfer of metabolites and regulatory molecules from one cell to another. Parietal granulosa cells, cumulus granulosa cells, and oocytes thus form a gap junction regulatory system. Intracellular exchange between oocytes and granulosa cells provides nutritional support for oocyte growth, while the health of the oocyte also simultaneously affects the condition of granulosa cells.

The luteinizing hormone (LH) peak before ovulation induces meiosis maturation *in vivo*. However, because the oocyte does not have a LH receptor, this induction maturation is mediated by granulosa cells in the follicle, indicating that cumulus cells are important for oocyte development. Importantly, oocytes also affect the differentiation and function of granulosa cells. Follicles without oocytes cannot develop, and studies have shown that in the absence of oocytes, CCs will not synthesize hyaluronic acid and therefore cannot be expanded *in vitro*^28,29^. Moreover, studies have shown that oocytes and granulosa cells are associated with oocyte-derived transforming growth factor-β members, such as growth and differentiation factor GDF9^30^. After gene manipulations in mice, GDF9 was found to be required for the formation of translocated zona pellucidas (TZPs), and the gene expression profiles of oocytes and granulosa cells showed significant differences in gene expression between the two cells. For example, oocyte-secreted factors, such as GDF9 and BMP15, are directly involved in the regulation of energy metabolism^31^ and cholesterol production^32^, and these pathways play roles in granulosa cells but not in oocytes. Therefore, these functions may be accomplished by oocyte paracrine factors acting on granulosa cells to meet the metabolic growth, maturation and embryogenesis requirements.

Mice in which the GDF9 gene is deleted exhibit female infertility due to a blockade of secondary follicle formation^30^, and BMP15 and GDF9 work synergistically in mice to maintain cumulus cell-oocyte complex (COC) integrity and female fertility^33^. As singletons, cattle have long breeding cycles, relatively low production efficiencies, and many reproductive diseases. Therefore, studying the mechanism of follicular development in bovine ovaries is important for improving the reproductive capacities of cattle.

In our previous study, we found that adding BMP15 and GDF9 to the CC growth medium significantly reduced CC apoptosis and promoted their proliferation. Moreover, adding both BMP15 and GDF9 simultaneously had an even larger effect, which was equal to that of adding a single agent alone when half of the concentration amount was added, thus showing that these two proteins have an additive effect. Therefore, this study aimed to investigate whether circRNAs are involved in regulation after *in vitro* addition of BMP15 and GDF9.

In recent years, non-coding RNAs, such as miRNAs, piRNAs, lncRNAs, and circRNAs, have been identified and considered to play important roles in organisms, thus becoming a research hotspot. Many studies have reported the roles of non-coding RNAs in animal reproduction. Murchison found that Dicer, a conserved RNase, regulates the biological origin of microRNAs and plays a key role in the regulation of mouse oocyte development^34^. Yan *et al*. found that miRNA-145 inhibits mouse granulosa cell apoptosis by targeting the activin receptor 1B^35^. Chen showed that miR-375 inhibits bovine CC proliferation and apoptosis by inhibiting BMPR2^36^. LncRNAs also play a vital role in reproduction. Han constructed the anterior pituitary lncRNA expression profiles of immature and mature rats and screened lncRNAs that affected the FSHβ gene. Therefore, we hypothesize that lncRNAs may regulate hormone secretion^37^.

With the rapid application of RNA sequencing technology and rapid development of biophysics, many exon transcripts have been shown to be reverse spliced into either non-linear or circular forms by gene rearrangement. In addition, circRNAs account for a significant proportion of all splicing transcripts. Jeck *et al*. detected up to 25,000 circRNAs in human fibroblasts^6^, and Memczak *et al*. identified 1950 human circRNAs, 1903 mouse circRNAs, and 724 circadian circRNAs using RNA-seq data in conjunction with the human leukocyte database^38^. As an important member of the non-coding RNA family, circRNAs have been extensively studied in human diseases^39,40^, and some studies have focused on the roles of circRNAs in animal reproduction. Dang reported the expression profiles of circRNAs in human pre-implantation embryos^41^, and Li studied the expression levels of circRNAs in mouse germline stem cells and the mechanisms underlying co-expression and ceRNA networks^42^. The study by Cheng aimed to investigate circRNA profiles in human granulosa cells during maternal aging and to uncover age-related circRNA variations that potentially reflect decreased oocyte competence^43^. However, few studies on circRNAs in cattle have been performed, especially in the field of breeding. In this study, we identified 1706 circRNAs transcripts from BMP15- and GDF9-treated CCs using the Illumina HiSeq Xten platform.

We analyzed the distribution, classification, and length characteristics of these circRNAs, and we identified the circRNAs differentially expressed in each treatment group. In addition, we performed KEGG and GO analyses of the source genes. Our results showed that BMP15 and GDF9 treatment altered the circRNA expression levels. However, only a small number of circRNAs was altered after the addition of BMP15 alone, whereas GDF9 alone and GDF9 in combination with BMP15 produced more circRNAs in CCs. From this result, we believe that BMP15 and GDF9 also have an additive effect on the effect of circRNAs and that BMP15 may play a role in assisting GDF9. According to GO analysis, the host genes of these circRNA are involved in biological processes, such as locomotion, reproduction, biological adhesion, growth, rhythmic processes, biological phases and hormone secretion. KEGG analysis showed that differentially expressed circRNAs related to multiple pathways, including thyroid hormone signaling pathway, ubiquinone, terpenoid-quinones, and tight junctions. These pathways can affect the proliferation and apoptosis of CCs, but further exploration is needed. These results indicate that circRNAs may play a broader role in the regulation of biological processes.

CircRNAs are a newly discovered type of non-coding RNA that has regulatory functions. Currently, circRNAs are known to function via the three following mechanisms: (1) regulating gene expression as miRNA sponges^44–47^; (2) regulating parental gene expression in cis; and (3) and forming complexes with proteins to perform biological functions. Currently, the most clearly elucidated of these three mechanisms is the miRNA sponge effect^1,5^.

To determine the effect of circRNAs on bovine CCs and the mechanism of ceRNAs, we screened all circRNAs that were consistently differentially expressed in all three treatment groups (BMP15, GDF9 and BMP15 + GDF9) (Fig. 2b). The expression levels of these three circRNAs were verified by qRT-PCR, and they all showed the same trend as that produced by RNA-seq (Fig. 4a) Circ_n/a_75 and circ_n/a_303, which originated from the intergenic region, were up-regulated in all three treatment groups, while circ_12691_1, derived from the exon region, was down-regulated in all three groups. These results also demonstrated the authenticity of our RNA-seq data.

We predicted circRNA-bound target miRNAs using RNAhybrid and Miranda software, and we mapped the circ_n/a_75 and circ_n/a_303 circRNA-miRNA co-expression network using Cytoscape.

We identified miR-339a as the target miRNA of circ_n/a_75 as well as miR-2400 and miR-30c as the target miRNAs of circ_n/a_303. We verified the expression levels of these three target miRNAs by qRT-PCR, and their expression levels in the treated groups were significantly lower than those in the control group. Correspondingly, the expression levels of circ_n/a_75 and circ_n/a_303 were up-regulated, which followed the trend (Fig. 4b). These data suggested that circRNAs may act as miRNA sponges by targeting miRNAs to inhibit their expression, thus regulating the expression of downstream target gene mRNAs. However, the specific mechanisms underlying this phenomenon need to be studied further.

Muroya *et al*. studied miRNAs associated with bovine mammary epithelial cells and found that miR-339a expression is decreased in prolactin-treated mammary epithelial cells^48^. Taxis found that miR-339a is associated with cattle challenged with bovine viral diarrhea virus^49^. Studies have found that miR-2400 promotes bovine preadipocyte proliferation^50^ and regulates the proliferation of skeletal muscle satellite cells by targeting the MYOG gene^51^. miR-30c has been shown to regulate cell proliferation and differentiation as well as to promote Schwann cell remyelination following peripheral nerve injury^52^. Furthermore, miR-30c may function in endometriosis by targeting plasminogen activator inhibitor-1^53^ and as a tumor suppressor via targeting SNAI1 in esophageal squamous cell carcinoma^54^. These miRNAs have been shown to play regulatory roles in cell proliferation and differentiation, but their roles in granulosa cells have not been elucidated and could be the focus of a follow-up study to investigate the effects of ceRNAs on bovine CCs.

In conclusion, for the first time, we constructed the circRNA expression profiles of BMP15 and GDF9 in CCs and preliminarily validated circ_n/a_75 and circ_n/a_303 as having miRNA sponge functions. These two circRNAs may regulate the additive effect of BMP15 and GDF9 and, thus, should be studied further.

## Materials and Methods

### Ethics statement

The experiments performed herein were in strict accordance to guidelines established by the Guide for the Care and Use of Laboratory Animals of Jilin University. In addition, all experimental protocols were approved by the Institutional Animal Care and Use Committee of Jilin University (permit number: 20160522).

### Cell culture and treatment

Healthy cow ovaries were collected from the No. 3 Slaughter House of the Haoyue Group in Changchun City, placed into a 37 °C thermos flask containing saline and penicillin-streptomycin, and shipped back to the laboratory within 30 min. The ovaries were cleaned with normal saline 3–5 times. Normal follicles (2–8 mm in diameter) were drawn from the ovary with a syringe to obtain follicular fluid, which was placed into a 50-ml centrifuge tube. COCs with more than 3 layers were selected, and tightly packed CCs and evenly distributed oocyte cytoplasms were verified under a microscope. COCs were cleaned 2–3 times, transferred to a hyaluronidase solution (0.1%) and repeatedly subjected to blowing pipette suction to completely separate the CCs from the oocytes and remove naked oocytes. This procedure was completed within 3 minutes. CCs in the hyaluronidase mixture solution were transferred to a centrifuge tube, and the blowing procedure was repeated. The CCs were evenly dispersed and then centrifuged at 1500 rpm. CCs were inoculated evenly on the cell culture plate, and the growth culture medium was replaced with a medium containing different cytokines when the cell density reached approximately 80%. The treatment conditions were as follows: a 4 nM HCL solution containing 1% BSA was added to the NC group; 100 ng/ml BMP15 (R&D, USA) was added to the BMP15 group; 200 ng/ml GDF9 (BioVision, USA) was added to the GDF9 group; and 50 ng/ml BMP15 and 100 ng/ml GDF9 were added to the BMP15 + GDF9 group. Incubation was continued at 37 °C in a 5% CO_2_ incubator for 48 h.

### Total RNA isolation and quality control

Total tissue RNA was extracted from each group using TRIzol (Invitrogen, NY, USA) according to the manufacturer’s recommended protocol. The qualities and concentrations of the RNA samples were determined using a NanoDrop ND-2000 spectrophotometer (NanoDrop Technologies). Genomic DNA (gDNA) contamination was excluded, and total RNA was purified by denaturing agarose gel electrophoresis. The samples were preserved at −80 °C for validation experiments.

### RNA library construction and sequencing

After passing the sample test, library construction was performed as follows:

Sample rRNA was removed using the Epicentre Ribo-ZeroTM Kit, and interfering rRNA was depleted with fragmentation buffer. The first cDNA strand was synthesized with random hexamers using the rRNA-depleted RNA as the template, and the second cDNA strand was synthesized by adding buffer, dATP, dUTP, dCTP, dGTP, RNase H and DNA polymerase I. cDNA was purified with AMPure XP beads, and purified double-stranded cDNA was then subjected to end-repair, ligated to the sequencing adapter, and size-selected with AMPure XP beads. Finally, the U chain was degraded, and a cDNA library was finally obtained by PCR enrichment. After library construction was completed, the quality of the library was tested to ensure that the requirements were met before being uploaded to the machine for testing. Quantitative quantification was performed using Qubit 2.0. The insert size of the library was tested using an Agilent 2100 Bioanalyzer to ensure that the insert size was as expected before proceeding to the next experiment. Q-PCR was used to assess the effective concentration for accurately quantifying the library (determined to be >2 nM) to complete the library test. After the inspection was completed, different libraries were pooled according to the target machine data volume and sequenced on the Illumina HiSeq platform by BioMarker Technologies (Beijing, China).

### Sequencing quality control and bioinformatics analysis

To ensure that the information was analyzed accurately, the original sequences that were obtained contained linker or low-quality sequences, and quality control protocols were applied to the original data to yield high-quality sequences (clean reads). We removed the reads containing the linker, and we filtered and removed low-quality data to ensure data quality. We also removed reads that contained more than 5% Ns (indeterminate base information). The clean data were aligned to the specified reference genome to obtain the mapped data. Based on the mapped data, the quality of the sequencing library was tested, assessing the insert lengths, randomness, CIRI prediction of circRNAs, circRNA-binding sites, circRNA gene analysis, expression analysis of different circRNA samples, GO analysis and KEGG enrichment of circRNA gene analysis.

### Identification of circRNAs

We used the CIRI and find_circ software packages to predict circRNAs separately. The CircBase database contains circRNA sequences from the following five species: humans, mice, nematodes, larva and coelacanths. Because our experimental samples were from cattle, CIRI software was used to predict the circRNAs. Because circRNA-looping sites cannot be aligned directly to the genome, find_circ^38^ uses the first 20 base pairs of each read end that are incompatible with the genome to anchor independent reads, thus matching the reference genome and finding only the matching site. The reference genome was the Bos taurus genomic sequence (version number is UMD3.1), which was downloaded from the Ensembl genome browser (http://www.ensembl.org/Bos_taurus/Info/Index). If the two anchors aligned in the linear region were in the reverse direction, the anchor reads were extended until the circRNA junction was found. The sequence was considered a circRNA if the two sides of the sequences corresponded to GT/AG splicing signals.

### Differential expression analysis

The amount of circRNA expression in each sample was calculated, and junction reads were used to indicate the amount of circRNA expression. The total productive maintenance (TPM) method was used for standardization. EBSeq was used to analyze the differential expression of circRNAs by assessing the difference between two samples expressing a CircRNA set. In the differential expression analysis of the circRNA test, FCs ≥ 2 and FDRs < 0.05 served as the screening criteria. The FC indicates the ratio of expression levels between two samples (groups). As the differential expression analysis of circRNAs is an independent statistical hypothesis test for many CircRNA expression levels, false-positive results are problematic. Thus, the well-known Benjamini-Hochberg calibration method was adopted for the analysis of the original P-values, and FDRs were used as the key screening indicator of differentially expressed circRNAs.

### Target site prediction and functional enrichment analysis

As circRNAs contain multiple miRNA-binding sites, miRNA target gene prediction methods can be used to identify miRNA-binding circRNAs and elucidate their functions based on functional annotations of the miRNA target genes. Herein, we used RNAhybrid and Miranda software. Host circRNA genes were used to list the gene names that were submitted to DAVID software for GO analysis^50^. KEGG enrichment analysis of the host circRNA genes was performed with KOBAS software^51^. Scores with P < 0.05 were considered significant for enrichment analysis.

### Quantitative real-time PCR analysis of circRNAs

The levels of circRNAs differentially expressed in the treatment groups were detected by real-time RT-PCR. The primer sequences for these circRNAs are listed in Supplementary Table S7. To determine the resistance of circRNAs to RNase R digestion, total RNAs were treated with RNase R (RNR-07250, Epicentre) prior to cDNA synthesis. Real-time PCR was performed using SYBR Green (Tiangen, China) according to the manufacturer’s protocol. The levels of circRNA expression were normalized to those of linear GAPDH. Real-time RT-PCR was conducted using the following reaction system: 10 μL of SYBR Premix DimerEraser (Tiangen, China), 1 μL of cDNA, 0.5 μL of the upstream and downstream primers, and 8 μL of RNase-free ddH2O. Real-time RT-PCR was performed with the following thermal cycling conditions: initial denaturation at 95 °C for 3 min, followed by 40 cycles of 95 °C for 30 s, 60 °C for 30 s, and 72 °C for 30 s. The circRNA expression levels were defined based on the threshold cycle (Ct), and relative expression levels were calculated via the 2−ΔΔCt method.

### CircRNA-miRNA co-expression network analysis

The circRNA-miRNA-mRNA co-expression network was built according to the predicted miRNA-binding sites, and analysis of interactions among this network was conducted using Cytoscape software. The sizes of the circles and triangles represent P-values, with larger sizes indicating smaller P-values.

### Statistical analysis

The experimental values are presented as the means ± SD of three independent experiments. Data were analyzed using SPSS 23.0. The significances of differences were determined by one-way ANOVA, and P < 0.05 was considered significant.

## Electronic supplementary material

Supplementary Information

Dataset 1
