# Supplementary material for: Genome-wide analysis of circular RNAs in bovine cumulus cells treated with BMP15 and GDF9
Source: Sci Rep. 2018 May 21;8:7944. doi: 10.1038/s41598-018-26157-2 (PMC5962577; doi:10.1038/s41598-018-26157-2)
Supplement: Supplementary file 1 — Supplementary Information [file 41598_2018_26157_MOESM1_ESM.pdf]

Genome-wide analysis of circular RNAs in bovine cumulus cells treated with BMP15 and GDF9

Yao Fu, Hao Jiang, Jian-Bo Liu, Xu-Lei Sun, Zhe Zhang, Sheng Li, Yan Gao, Bao Yuan*, Jia-Bao Zhang*

Department of Laboratory Animal Science, College of Animal Sciences, Jilin University, Changchun 130062, Jilin, P.R. China

***Correspondence:**

Jia-Bao Zhang

E-mail: zjb515@126.com

Tel.: +86-431-8783-6551

Bao Yuan

E-mail: yuan_bao@jlu.edu.cn

Tel.: +86-431-8783-6536

Supplementary Figures

S1

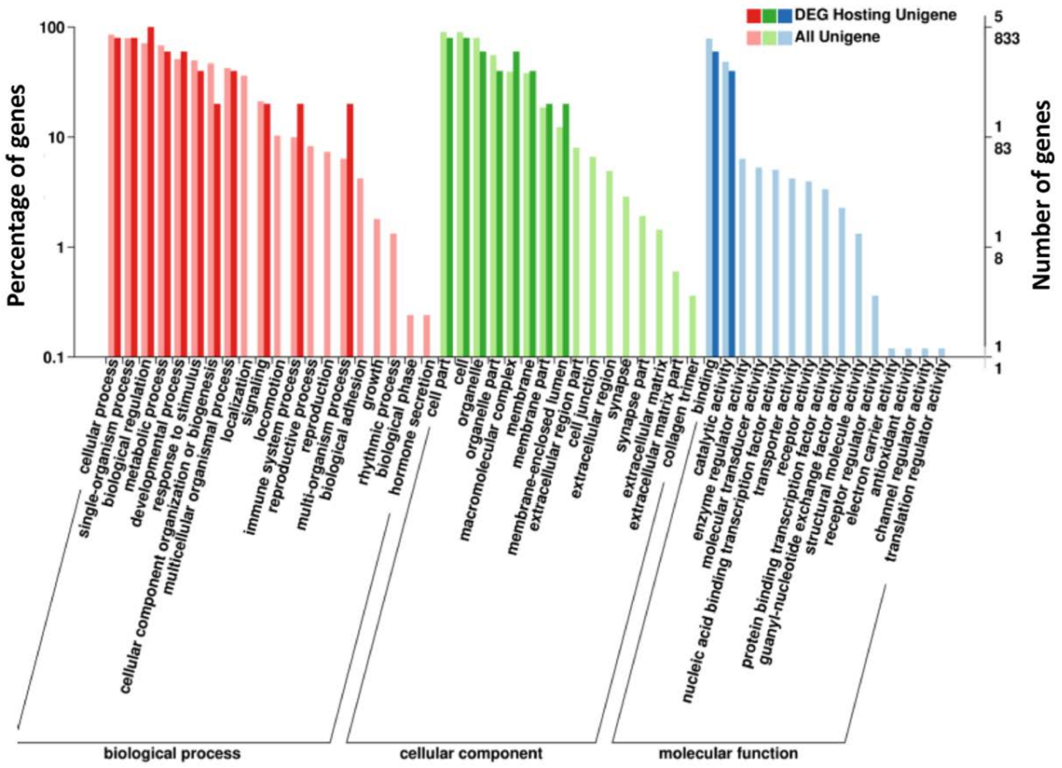

Supplemental Fig. S1 GO annotations of host genes of the differential circRNAs between NC group and BMP15 group.

S2

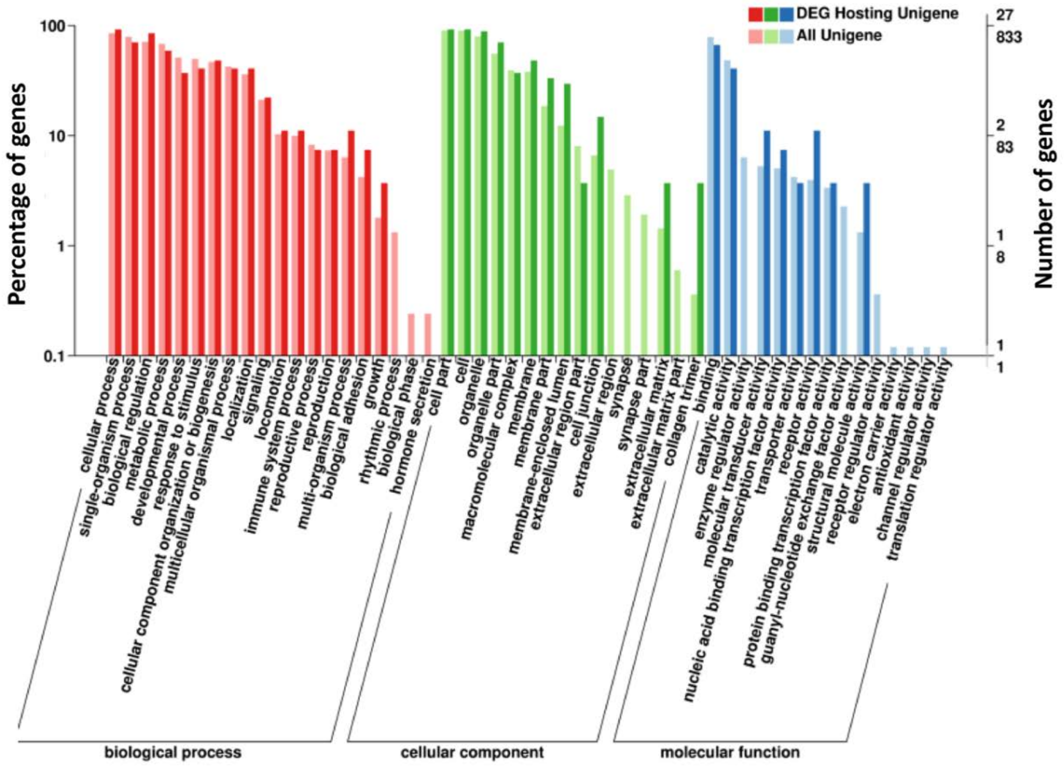

Supplemental Fig. S2 **GO annotations of host genes of the differential circRNAs between NC group and GDF9 group.**

S3

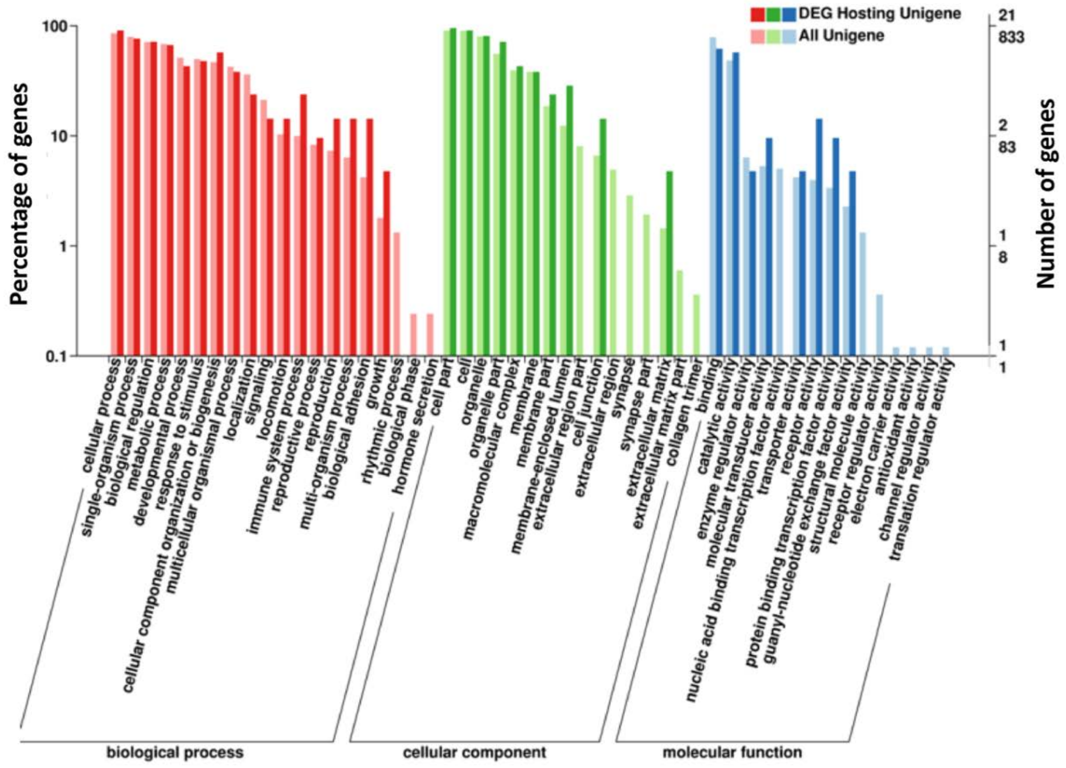

Supplemental Fig. S3 GO annotations of host genes of the differential circRNAs between NC group and BMP15+GDF9 group.

Supplementary Tables

Table S1. Differentially expressed circRNAs between NC group and BMP15 group.

#ID	NAME	NC_TPM	BMP15_TPM	FDR	log2FC	regulated
12:26345626|26375936	n/a_75	0.000	89.502	0.027	4.632	up
13:47225429|47256127	n/a_97	46.415	0.000	0.048	-3.815	down
16:44322285|44332990	ENSBTAG10401_1	3207.544	0.000	0.030	-4.217	down
18:22284519|22302243	ENSBTAG12501_1	0.000	6425.864	0.036	4.350	up
23:24202293|24204665	ENSBTAG11237_7	3706.495	0.000	0.043	-3.915	down
27:25868033|25879425	ENSBTAG12691_1	5836.182	0.000	0.023	-4.434	down
29:37010118|37011191	n/a_272	1785.951	0.000	0.030	-4.217	down
5:34140527|34156259	n/a_303	0.000	137.307	0.037	4.318	up
9:63144654|63146741	ENSBTAG03764_1	5032.945	0.000	0.046	-3.866	down

Table S2. Differentially expressed circRNAs between NC group and GDF9 group.

#ID	NAME	NC_TPM	GDF9_TPM	FDR	log2FC	regulated
10:3249430|3249848	ENSBTAG11076_1	3400.674	0.000	0.028	-4.047	down
12:26345626|26375936	n/a_75	0.000	58.523	0.039	4.341	up
13:33503657|33505350	ENSBTAG01552_1	1902.888	0.000	0.044	-3.694	down
13:52644069|52645696	ENSBTAG11425_1	5184.084	0.000	0.025	-4.148	down
16:34260887|34298462	ENSBTAG17788_1	2861.785	0.000	0.044	-3.694	down
16:38409896|38463364	ENSBTAG17727_1	1386.817	0.000	0.024	-4.195	down
18:1784918|1807715	n/a_151	0.000	66.952	0.048	4.135	up
18:35671821|35692167	ENSBTAG04368_2	1829.211	0.000	0.009	-4.948	down
2:118712720|118729424	n/a_189	88.577	0.000	0.027	-4.098	down
21:2404179|2410197	n/a_204	0.000	411.229	0.025	4.801	up
22:8132547|8140204	ENSBTAG17426_2	0.000	3928.870	0.032	4.548	up
23:8642065|8647992	ENSBTAG34493_1	0.000	4557.712	0.045	4.207	up
23:23987169|24010090	ENSBTAG11237_2	2504.187	0.000	0.028	-4.047	down
23:24122351|24182398	ENSBTAG11237_4	0.000	2271.378	0.048	4.135	up
24:934078|947669	n/a_233	96.768	0.000	0.033	-3.939	down
24:42351861|42355476	ENSBTAG17279_1	8755.537	0.000	0.008	-5.055	down
25:39188874|39198380	ENSBTAG17175_2	8220.476	0.000	0.006	-5.272	down
26:41878873|41902762	ENSBTAG14064_1	2144.099	0.000	0.030	-3.994	down
27:15583487|15586769	ENSBTAG20657_2	0.000	615.719	0.033	4.520	up
27:25868033|25879425	ENSBTAG12691_1	5836.182	0.000	0.013	-4.672	down

27:26117250|26128053	n/a_259	106.522	0.000	0.040	-3.759	down
29:1011931|1015558	ENSBTAG03552_4	1799.776	0.000	0.044	-3.694	down
3:29541834|29552476	ENSBTAG11322_1	3373.906	0.000	0.004	-5.498	down
3:30886834|30892695	ENSBTAG14294_2	2115.564	0.000	0.040	-3.759	down
3:58920288|58930226	ENSBTAG34776_1	3728.107	0.000	0.044	-3.694	down
3:67179509|67187976	ENSBTAG20761_1	1712.599	0.000	0.033	-3.939	down
3:75198283|75214573	ENSBTAG15482_2	2057.827	0.000	0.047	-3.626	down
3:121133595|121136000	ENSBTAG13253_1	3956.379	0.000	0.027	-4.098	down
5:34140527|34156259	n/a_303	0.000	110.127	0.041	4.308	up
5:51300530|51305147	ENSBTAG11068_1	0.000	7130.172	0.016	5.272	up
7:17574398|17574848	ENSBTAG18894_1	2916.355	0.000	0.033	-3.939	down
7:48437904|48438652	ENSBTAG16105_1	7726.349	0.000	0.012	-4.738	down
7:97238192|97242253	ENSBTAG09131_1	0.000	3426.985	0.030	4.629	up
8:78506771|78524362	n/a_378	0.000	110.215	0.035	4.463	up
9:51063554|51072760	ENSBTAG19729_1	0.000	3708.147	0.028	4.705	up
X:14211352|14221024	ENSBTAG16263_1	2450.024	0.000	0.047	-3.626	down

Table S3. Differentially expressed circRNAs between NC group and BMP15+GDF9 group.

#ID	NAME	NC_TPM	BMP15+GDF9_TPM	FDR	log2FC	regulated
12:26345626|26375936	n/a_75	0.000	56.098	0.037	4.212	up
14:3934740|3960034	ENSBTAG09578_1	0.000	3899.146	0.032	4.349	up
18:15963492|15984525	ENSBTAG04806_1	3405.246	0.000	0.027	-4.180	down
19:15415228|15416261	ENSBTAG18689_1	0.000	3782.988	0.041	4.100	up
2:118712720|118729424	n/a_189	88.577	0.000	0.036	-3.949	down
20:6614334|6618392	ENSBTAG16002_1	3001.391	0.000	0.036	-3.949	down
21:66118264|66125084	ENSBTAG18310_1	0.000	2099.582	0.047	3.978	up
22:8132547|8140204	ENSBTAG17426_2	0.000	2727.457	0.047	3.978	up
23:7083607|7105859	ENSBTAG03038_1	0.000	120.651	0.019	4.842	up
23:8642065|8647992	ENSBTAG34493_1	0.000	5070.521	0.035	4.282	up
23:23987169|24010090	ENSBTAG11237_2	2504.187	0.000	0.038	-3.898	down
23:24194410|24204665	ENSBTAG11237_6	0.000	1449.496	0.049	3.935	up
23:24202293|24204665	ENSBTAG11237_7	3706.495	0.000	0.034	-3.998	down
26:41878873|41902762	ENSBTAG14064_1	2144.099	0.000	0.040	-3.846	down
27:15583487|15586769	ENSBTAG20657_2	0.000	885.951	0.017	4.953	up
27:25868033|25879425	ENSBTAG12691_1	5836.182	0.000	0.018	-4.520	down
27:42256612|42288996	ENSBTAG47930_1	0.000	5095.783	0.047	3.978	up
28:24649582|24683148	ENSBTAG14029_1	2210.330	0.000	0.025	-4.262	down
29:1014460|1015558	ENSBTAG03552_5	3540.257	0.000	0.040	-3.846	down

3:58688495|58692244	n/a_280	657.638	0.000	0.016	-4.648	down
3:67179509|67187976	ENSBTAG20761_1	1712.599	0.000	0.043	-3.791	down
5:34140527|34156259	n/a_303	0.000	136.520	0.027	4.532	up
6:43494466|43531421	n/a_320	0.000	37.536	0.049	3.935	up
7:24005619|24024743	ENSBTAG25443_2	0.000	1866.369	0.033	4.316	up
7:48437904|48438652	ENSBTAG16105_1	7726.349	0.000	0.017	-4.585	down
7:97238192|97242253	ENSBTAG09131_1	0.000	2665.202	0.037	4.212	up
9:51063554|51072760	ENSBTAG19729_1	0.000	3519.454	0.026	4.561	up
9:63144654|63146741	ENSBTAG03764_1	5032.945	0.000	0.036	-3.949	down

Table S4. KEGG enrichment of circRNA host genes between NC group and BMP15 group.

#Pathway	ko_ID	Corrected_P-value	Gene ID
Protein processing in endoplasmic reticulum	ko04141	0.037	ENSBTAG00000010401
Viral carcinogenesis	ko05203	0.058	ENSBTAG00000012691

Table S5. KEGG enrichment of circRNA host genes between NC group and GDF9 group.

#Pathway	ko_ID	Corrected_P-value	Gene ID
Ubiquinone and other terpenoid-quinone biosynthesis	ko00130	0.087	ENSBTAG00000019729
Systemic lupus erythematosus	ko05322	0.044	ENSBTAG00000016105
Central carbon metabolism in cancer	ko05230	0.044	ENSBTAG00000014064
Tight junction	ko04530	0.205	ENSBTAG00000017279
RNA degradation	ko03018	0.275	ENSBTAG00000009131
Peroxisome	ko04146	0.044	ENSBTAG00000018894

Thyroid hormone signaling pathway	ko04919	0.205	ENSBTAG00000003552
Viral carcinogenesis	ko05203	0.308	ENSBTAG00000012691
Bile secretion	ko04976	0.044	ENSBTAG00000011076
Choline metabolism in cancer	ko05231	0.340	ENSBTAG00000017788
Endocytosis	ko04144	0.370	ENSBTAG00000017426
HTLV-I infection	ko05166	0.167	ENSBTAG00000004368

Table S6. KEGG enrichment of circRNA host genes between NC group and BMP15+GDF9 group.

#Pathway	ko_ID	Corrected_P-value	Gene ID
RNA degradation	ko03018	0.295	ENSBTAG00000009131
Primary immunodeficiency	ko05340	0.048	ENSBTAG00000003038
Central carbon metabolism in cancer	ko05230	0.048	ENSBTAG00000014064
Systemic lupus erythematosus	ko05322	0.048	ENSBTAG00000016105
Base excision repair	ko03410	0.094	ENSBTAG00000018689
Thyroid hormone signaling pathway	ko04919	0.220	ENSBTAG00000003552

Ubiquinone and other terpenoid-quinone biosynthesis	ko00130	0.094	ENSBTAG00000019729
Endocytosis	ko04144	0.395	ENSBTAG00000017426
Protein processing in endoplasmic reticulum	ko04141	0.220	ENSBTAG00000047930
Insulin signaling pathway	ko04910	0.138	ENSBTAG00000004806
Ubiquitin mediated proteolysis	ko04120	0.330	ENSBTAG00000014029
Viral carcinogenesis	ko05203	0.330	ENSBTAG00000012691
Small cell lung cancer	ko05222	0.220	ENSBTAG00000009578

Table S7. Primer sequences for circRNAs

circRNA	Sense（5'-3'）	Antisense（5'-3'）
**circ_n / a_75**	ACACCGAATTTCACTGTTTCTCC	GGCAATTGTTTGATCGTTACTGG
**circ_12691_1**	CGCTGATGCCGTGTGTTTAC	GCGAGGCTTAGGAGGGATTC
**circ_n / a_303**	TGGCAAACACTGGAATCCTACT	CACAAGCTTGCAAATGAAGGA
